# Clinical Presentation of a Patient with Localized Acquired Cutis Laxa of Abdomen: A Case Report

**DOI:** 10.1155/2010/402093

**Published:** 2010-03-04

**Authors:** Tugomir Gverić, Marko Barić, Vedrana Bulat, Mirna Šitum, Jana Pušić, Dubravko Huljev, Boris Zdilar, Snježana Gverić-Ahmetašević, Davor Tomas

**Affiliations:** ^1^Department of Plastic and Reconstructive Surgery, Clinical Hospital “Sveti Duh”, Zagreb, Croatia; ^2^Department of Dermatology and Venereology, University Hospital “Sestre milosrdnice”, Zagreb, Croatia; ^3^General Practice, Health Care Center “dr. Gopčević”, Zagreb, Croatia; ^4^Department of Perinatology and Intensive Care, Clinical Hospital Center Zagreb, University Hospital for Obstetrics and Gynaecology, Petrova, Zagreb, Croatia; ^5^Department of Pathology, University Hospital “Sestre milosrdnice”, Zagreb, Croatia

## Abstract

*Background*. Cutis laxa (CL) is a rare disorder of elastic tissue characterized by loose, sagging skin with reduced elasticity, and resilience without resulting scarring. CL may be inherited as a dominant, recessive, or X-linked recessive disease, or acquired. The heritable forms of CL predominantly begin at birth, but it may be delayed until puberty or age of 30 years with extracutaneous manifestations including pulmonary emphysema, umbilical and inguinal hernias, and gastrointestinal and vesicourinary tract diverticuli. An acquired form of the disease occurs in adults with no evidence of internal organ involvement. *Objective*. The aim of this case report was to present our patient suffering from CL, and to evaluate clinical presentation, diagnostic and therapeutic difficulties in this rare condition. *Case Report*. A 30-year-old female patient was admitted to our Hospital due to localized loose and sagging skin of abdomen, induced by prior cesarean section 6 years ago. CL has been diagnosed based on the clinical picture and pathohistological appearance. *Conclusion*. Reconstructive surgery provides a dramatic cosmetic improvement with significant psychosocial benefit. Repeated surgical procedures may be required to correct the lax skin, which worsens with age.

## 1. Introduction

Cutis laxa (synonym: dermatochalasia, dermatomegaly) (CL) is a rare connective tissue disorder caused by defects in the elastic fiber network and can affect multiple tissues, predominantly the skin [1]. There are no accurate incidence and prevalence data available. Approximately 35 cases of CL have been reported to date in the literature. There is no racial or ethnic predilection. CL affects both sexes equally. The clinical presentation and the mode of inheritance show considerable heterogeneity. It may be inherited as a dominant, recessive, or X-linked recessive disease or acquired. The congenital forms of CL primarily affect newborns with associated extracutaneous manifestations including pulmonary emphysema, umbilical and inguinal hernias and gastrointestinal and vesico-urinary tract diverticuli that can cause considerable morbidity and mortality during infancy [2]. An acquired form of the disease may develop at any age, but usually occurs in second and third decade of life, with no evidence of internal organ involvement. Acquired form of CL has a benign course, although some cases with systemic involvement were reported [3–5]. 

 Generalized skin involvement is most commonly observed in acquired CL and localized form of CL is extremely rarely observed. Acquired CL may occur without an identifiable cause, present as a paraneoplastic phenomenon (lymphoma, multiple myeloma), or follow prior inflammation of the skin. It was also described within the ensuing weeks or months after allergic reactions, arthropod stings (Borrelia burgdorferi) or CL can be drug-induced (D-penicillamine, isoniazid) According to some authors CL can be associated with systemic lupus erythematosus, rheumatoid arthritis, amyloidosis, sarcoidosis, nephrotic syndrome, celiac disease, syphilis and alpha -1 antitrypsin deficiency. The etiopathology of CL is still not completely understood but recent electron microscopic studies have suggested that there is a loss and fragmentation of elastic fibers in the skin, thus leading to loose, sagging skin with reduced elasticity and resilience without resulting scarring because collagen fibers are usually not affected. The enhanced degradation of the elastic fibers is a possible mechanism for the development of acquired localized CL due to powerful proteolytic elastases which are released from inflammatory cells, such as polymorphonuclear leukocytes and monocyte-macrophages into extracellular matrix [6]. Furthermore, immunopathogenetic mechanisms may play a role in a small subset of patients, as evidenced by IgG and IgA deposits in lesional skin or paraproteinemia [7]. There may be an underlying genetic susceptibility for the development of acquired CL, for example, missense alleles in the elastin and fibulin-5-genes [8, 9]. Diagnosis of CL is supported by histopathologic analysis of lax skin and the lack or shortening of elastic fibers (orcein stain). In our case of late-onset CL, the elastic fibers were diminished. Electron microscopy confirms an irregular fragmentation of the fiber structure [10]. The collagen, however, within the dermis of affected skin appears normal. Definite diagnosis of localized acquired CL is sometimes difficult to make since elastic fibers cannot be identified in routinely fixed and stained histologic sections. Thus, it is essential to analyze histopathologic findings of the affected tissue by the experienced pathologist. Management of skin lesions is preferentially conducted by a plastic surgeon. Since localized acquired CL can produce significant disfigurement to patients, reconstructive surgical treatment is indicated [11, 12]. However, disease may reoccur, necessitating additional surgical procedures. The purpose of this case report was to present our patient suffering from CL, and to evaluate clinical presentation, diagnostic and therapeutic difficulties in this rare condition.

## 2. Case Report

**Figure 1:**
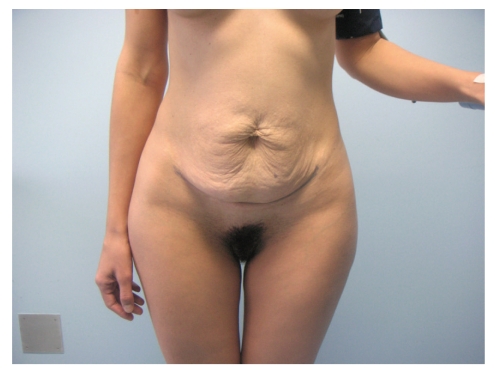
Anteroposterior view of a 30-year-old female patient with Cutis laxa before abdominoplasty.

**Figure 2:**
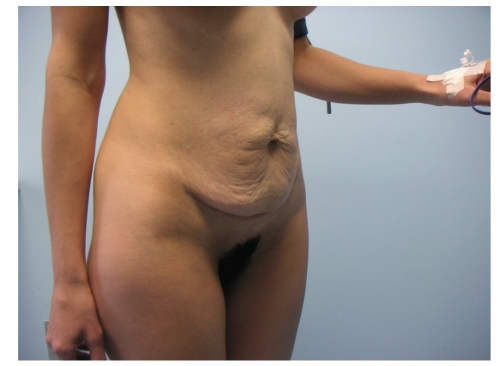
Lateral view of a 30-year-old female patient with Cutis laxa before abdominoplasty.

A 30-year-old Caucasian female patient was admitted to our Hospital in February 2009, due to prominent, localized loose and sagging skin of abdomen, with reduced elasticity and resilience which gave her prematurely aged appearance induced by prior cesarean section 6 years ago (Figures 1 and 2). One month after cesarean section due to prolonged labor, she noticed the development of loose, wrinkled skin of abdomen without any sign of inflammation. The patient was treated by her gynaecologist with topical emollients, but with no result. At that time, patient refused reconstructive surgery of the redundant abdominal skin and had second cesarean section 3 years ago due to prolonged labor. 

 On admission to our Hospital her height was 175 centimeters and weight was 60 kilograms, body mass index was normal (19,6). Lax, redundant folds in the lower part of the abdominal wall were not associated with papules or plaques and were on non-sun-exposed areas.The affected area has normal pigmentation and no associated scaling. There was a lack of cutaneous induration or sclerosis. Skin lesions were asymptomatic and lack clinical sings of inflammation. Loss of recoil due to impaired function of the elastic fiber network has led to pendulous skin which was most notable in abdomen. These findings were limited to the skin of abdomen and were primarily of major cosmetic concern to patient. Extracutaneous findings were absent. She had had no previous illnesses and without any subjective difficulties (e.g., pain, fever, weight loss). There was no family history of this disorder and skin lesions were not present at birth and developed during 2nd decade of life. There was no mucosal involvement. She was not taking any medication. History of chronic sun exposure was negative. She was non-smoker. 

**Figure 3:**
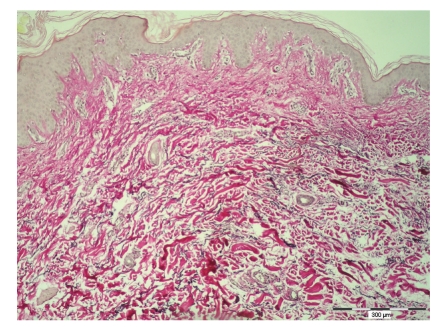
Pathohistological analysis of the skin showed loss of elastic tissue in the dermis and the remaining fibers were disorganized, shortened and fragmented (Orcein, bar = 300 *μ*m).

 CL has been diagnosed based on the clinical picture and pathohistological appearance. On admission excisional biopsy of lax skin has been performed. Routine hematoxylin-eosin staining has revealed normal collagen fibers, without any inflammatory infiltrate. Histopathologic analysis of skin has shown characteristic histologic feature of CL such as a loss of dermal elastic fibers revealed by orcein stain. Diminished elastic tissue was revealed throughout the dermis, not extending into the subcutis. The remaining elastic fibers were disorganized, shortened and fragmented. There was no epidermal alterations or cellular infiltrate (Figure 3). 

**Figure 4:**
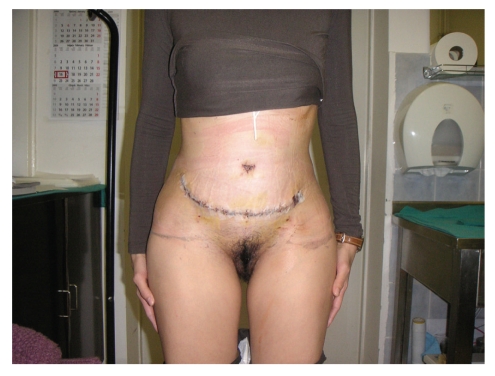
Post operative anteroposterior view.

**Figure 5:**
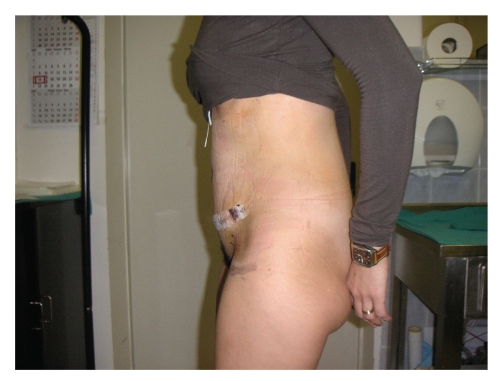
Post operative lateral view.

 There was no evidence of IgG and IgA deposits in lesional skin.The following diagnostic and laboratory tests have been performed during hospitalization. Obtained findings include the erythrocyte sedimentation rate, complete blood count with differential and platelet count—values were within normal limits. The following tests were normal as well (glucose, transaminases, electrophoresis, immunoelectrophoresis, C3, C4, ANA, bilirubin, total protein, blood urea nitrogen, creatinine, electrolytes, iron, cholesterol and triglyceride levels, urine analysis). Other laboratory findings were also regular (pharyngeal and nasal swabs, oral cavity mycological analysis, PPD, hemoccult test). A basic laboratory assessment of clotting was performed including a platelet count, prothrombin time, and activated partial thromboplastin time. Obtained echocardiogram and abdominal ultrasound were without significant changes. There were no radiological signs for pulmonary emphysema. Serum level of *α*_1_-antitrypsin and tumor markers (CA 19-9, CEA, CA 125, CA 15-3, and CYFRA 21-1) was normal as well. A serum thyroid-stimulating hormone (TSH) level was normal. Additional laboratory findings include free T3 and free T4 levels, and antithyroperoxidase and antithyroglobulin antibodies. Patient was sent to a plastic surgeon to undergo abdominoplasty and reduction of subcutaneous fat (Figures 4 and 5). Wound healing was normal and there was no clinical sign of dehiscence.

## 3. Discussion

Localized acquired CL is extremely rare connective tissue disorder. The overall incidence and prevalence of CL remain unknown for several possible reasons. There are no epidemiologic databases for this entity; most people do not present to a physician for this condition or the disease may be unrecognized by physician. In the case of our patient, several questions arose regarding the cause, localization and course of the disease. Localized forms of acquired CL have been described in the literature with few cases localized on the face, but there are no data available for CL that involves only the skin of the abdomen [13, 14]. There are a number of the conditions, both inherited and acquired, which have similarities to acquired CL and must be considered in the differential diagnosis. Very common finding of redundant skin folds after pregnancy is most commonly associated with striae distensae, erythematous or blue-red elevated lesions on lateral parts of abdomen and breasts. In contrast to redundant skin folds after pregnancy, our patient did not show signs of striae distensae. Diffuse wrinkling of the skin in our patient did not disappear when we stretched the skin in contrast to redundant skin folds after pregnancy. In Ehlers-Danlos syndrome, heritable disorder in various collagens, the skin is hyperextensible, but still displays normal recoil. A group of authors have reported localized, acquired CL confined to sun-exposed areas such as around the eyes, the face, the neck, and the shoulders which has progressed despite surgical procedures [15]. It is well known that UV irradiation and skin aging induces subsequent degradation of the extracellular matrix, but in the case of our young patient skin lesion was confined to non-sun-exposed area. In our case, as it is common for the acquired form of CL, skin lesion appeared during 2nd decade of life. Initial assessment should include histological examination of skin lesion. Histopathologic analysis of skin lesion has shown characteristic histologic feature of CL such as a loss of dermal elastic fibers revealed by orcein stain and normal collagen fibers. 

 Diminished elastic tissue was revealed throughout the dermis, not extending into the subcutis. Mid-dermal elastolysis must be differentiated from CL while in this disorder of elastic fibers diminished elastic tissue is revealed in mid-dermis, even though those in the papillary and deeper dermis are normal. Examining the dermo-epidermal junction the oxytalanic fibers in the papillary dermis were affected, ranging from reduction to absence giving an irregular contour the basal membrane. Definite diagnosis of localized acquired CL is sometimes difficult to make since elastic fibers cannot be identified in routinely fixed and stained histologic sections. Occasionally a scant, but not diagnostic, perivascular lymphohistiocytic infiltrate is seen. There was no epidermal alteration or cellular inflammatory infiltrate and also no evidence of IgG and IgA deposits in lesional skin which could, as in the case report of acquired CL described by Krajnc et al., lead to conclusion that, in our case, immunopathogenetic mechanisms may not play a role in the loss of elastin fibres [7]. 

 In this case, the histopathologic analysis did not provide clues of true secondary causes. 

 According to De Almeida et al. acquired CL was also described within the ensuing weeks or months after allergic reactions, arthropod stings or CL can be drug-induced (D-penicillamine, isoniazid) but there was neither medical history of taking any medications nor allergic reactions and arthropod stings [12]. Since this skin lesion in our patient has manifested after two cesarean sections due to prolonged labor, increased physical stress on the elastic fiber network, as is common for skin of abdomen in pregnant women, may be possible explanation of the loss of elastic fibers. 

 The treatment of CL is generally only symptomatic. Reconstructive surgery of the redundant abdominal skin provides a dramatic cosmetic improvement with significant psychosocial benefit, in accordance to previous reports of the surgical plastic procedure [16]. 

 Unfortunately, repeated surgical procedures may be required to correct the lax skin, which worsens with age.

## 4. Conclusion

Localized acquired CL poses a clinical challenge to the dermatologist due to its rarity, complexity and variability of the phenotypic manifestations. Our patient presented with an atypical localization of CL. This can be explained by increased physical stress on the elastic fiber network due to prolonged labors. Definite diagnosis of localized acquire CL is sometimes difficult to make because elastic fibers cannot be identified in routinely fixed and stained histologic sections. Thus, it is essential to analyze the clinical presentation, histopathologic findings of the affected tissue, laboratory results, and sometimes electron immunomicroscopy. 

 Currently, no specific treatment is available for CL. Although localized CL has no medical consequences, it can frequently produce significant disfigurement to those afflicted. Reconstructive surgery of the redundant abdominal skin provides a dramatic cosmetic improvement with significant psychosocial benefit.

## References

[1] Ringpfeil F, Uitto J, Bolognia JL, Jorizzo JL, Rapini RP (2008). Heritable disorders of connective tissue. *Dermatology*.

[2] Urban Z, Gao J, Pope FM, Davis EC (2005). Autosomal dominant cutis laxa with severe lung disease: synthesis and matrix deposition of mutant tropoelastin. *Journal of Investigative Dermatology*.

[3] Lewis KG, Bercovitch L, Dill SW, Robinson-Bostom L (2004). Acquired disorders of elastic tissue: part II. decreased elastic tissue. *Journal of the American Academy of Dermatology*.

[4] Tan S, Pon K, Bargman J, Ghazarian D (2003). Generalized cutis laxa associated with heavy chain deposition disease. *Journal of Cutaneous Medicine and Surgery*.

[5] Braverman IM (1998). *Skin Signs of Systemic Diseases*.

[6] Shapiro SD (1998). Matrix metalloproteinase degradation of extracellular matrix: biological consequences. *Current Opinion in Cell Biology*.

[7] Krajnc I, Rems D, Vizjak A, Hödl S (1996). Acquired generalized cutis laxa with monoclonal gammopathy (IgG lambda). Clinical, histological and immunofluorescence evaluation and a review of the literature. *Hautarzt*.

[8] Hu Q, Reymond J-L, Pinel N, Zabot M-T, Urban Z (2006). Inflammatory destruction of elastic fibers in acquired cutis laxa is associated with missense alleles in the elastin and fibulin-5 genes. *Journal of Investigative Dermatology*.

[9] Yanagisawa H, Davist EC, Starcher BC (2002). Fibulin-5 is an elastin-binding protein essential for elastic fibre development in vivo. *Nature*.

[10] Hucthagowder V, Sausgruber N, Kim KH, Angle B, Marmorstein LY, Urban Z (2006). Fibulin-4: a novel gene for an autosomal recessive cutis laxa syndrome. *American Journal of Human Genetics*.

[11] Hill VA, Seymour CA, Mortimer PS (2000). Penicillamine-induced elastosis perforans serpiginosa and cutis laxa in Wilson’s disease. *British Journal of Dermatology*.

[12] De Almeida HL, Da Rocha MP, Neugebauer S, Wolter M, Rocha NM (2007). Acquired cephalic cutis laxa. *Dermatology Online Journal*.

[13] Dózsa A, Károlyi ZS, Degrell P (2005). Bilateral blepharochalasis. *Journal of the European Academy of Dermatology and Venereology*.

[14] Riveros CJP, Bejarano Gavilán MF, Franca LFS, Sotto MN, Takahashi MDF (2004). Acquired localized cutis laxa confined to the face: case report and review of the literature. *International Journal of Dermatology*.

[15] Filippopoulos T, Paula JS, Torun N, Hatton MP, Pasquale LR, Grosskreutz CL (2008). Periorbital changes associated with topical bimatoprost. *Ophthalmic Plastic and Reconstructive Surgery*.

[16] Wong MC, Georgeu GA, Sassoon EM, O’Neill TJ (2002). A case report of cutis laxa in one of identical twins. *Aesthetic Plastic Surgery*.

